# The impact of information literacy and environmental factors on innovation performance in teacher education: the mediating role of quantity and quality oriented online knowledge sharing process

**DOI:** 10.3389/fpsyg.2026.1887891

**Published:** 2026-08-18

**Authors:** Asil Derin

**Affiliations:** Faculty of Education, Kafkas University, Kars, Türkiye

**Keywords:** Biggs’s Presage-Process-Product model, information literacy, innovation performance, online knowledge sharing process, structural equation modeling, teacher education

## Abstract

This study, based on Biggs’s Presage-Process-Product model, examined the direct and indirect paths of the relationship between information literacy, environmental factors, online knowledge sharing process, and innovation performance by using structural equation modeling (SEM) and correlational survey model. This study was conducted with a total of 503 pre-service teachers, of whom 302 were female and 201 were male. As a result, information acquisition, awareness, discrimination, application literacy, and environmental factors related to online learning platforms were found to positively predict innovation performance. Compared to information application literacy, information awareness and discrimination literacy had a more significant effect on innovation performance. In addition, environmental factors (such as peer and platform support) had a significant positive impact on innovation performance. Finally, when compared to the Qty-KSP (quantity-oriented online knowledge sharing process) variable, Qlty-KSP (quality-oriented online knowledge sharing process) was a stronger mediating variable and played a positive mediating role in the effect of information literacy and environmental factors on innovation performance. The findings highlighted the importance of pre-service teachers’ innovation performance in promoting the sustainable development of higher education. This could be achieved by enhancing pre-service teachers’ information literacy and ensuring the quality of the online learning process.

## 1 Introduction

According to Evgeny Mozorov, a researcher and writer who focuses on the political and social applications of technology, “innovation” is generally linked to the rise of new technologies, economic rationalism, and a phenomenon*“fetishization of innovation”* (Snyder, 1998). In teacher education, innovation serves the process of change when new technological products (or solutions) are developed (for example, the use of computer game-like simulations to prepare newly hired teachers for behavior management) or competition is encouraged (for example, allowing private universities to compete with public universities) (Mozorov, 2014). Just like in health, information technology, and technology, the field of education is witnessing new developments every day. Therefore, it is of great importance for teachers to be open to these innovations and willing to implement them (Baysen et al., 2017). Various factors (such as technological advancements, changing expectations regarding education, the diversity of student profiles and needs, traditional educational approaches are no longer accepted today, and the use of new, effective, and constructivist teaching and learning methods) make it essential for today’s teachers to adopt innovative approaches (West and Farr, 1990). Innovative teachers are able to develop themselves, employ current and unique approaches in delivering information. Moreover, they can ensure students’ active participation in the learning process, and implement new knowledge and skills by blending various methods to enhance and sustain this participation (Ritchhart, 2004).

As can be understood, it is believed that teachers with innovative qualities have a responsibility to keep up with current changes and developments in the field of education. In fulfilling these responsibilities, it is of great importance for teachers to be equipped with information literacy as the requirements of the current era (Kurbanoğlu and Akkoyunlu, 2008). As an important component of 21st century higher education, there is a great interest to teach information literacy skills to university students. Furthermore, it is particularly important to teach these skills to pre-service teachers. In this way, they can effectively teach these skills to their future students and act as effective role models (O’Hanlon, 1987; Witt, 2024). Indeed, the relevant literature indicates that teachers with high level of information literacy skills can develop themselves and contribute to the establishment of appropriate learning environments. Accordingly, they can develop information literacy skills of their students (Johnson and O’English, 2004; Kurbanoğlu and Akkoyunlu, 2008). Therefore, in the 21st century, teachers play a key role in raising individuals with the information literacy skills required by society. But it is essential that pre-service teachers are equipped with these skills before they begin teaching.

It can be said that information literacy consists of the skills to access, evaluate, organize, and apply information from various sources (Humes, 2003, as cited in Akkoyunlu and Yılmaz, 2005). Furthermore, in order for individuals to stay afloat in the rapidly enlarging pool of information, they must have the skills to analyze knowledge needs, identify various types of sources, evaluate tools for accessing information, and reorganize information (Laverty, 1997, as cited in Akkoyunlu and Yılmaz, 2005). In other words, individuals equipped with information literacy skills are also individuals who have learned how to learn (Akkoyunlu and Yılmaz, 2005). In this context, the characteristics of individuals equipped with information literacy skills may be summarized as follows: They are able to recognize accurate and comprehensive information, identify their knowledge needs, formulate these needs by asking questions, identify potential information sources, develop effective strategies for information seeking, access information sources using computers and other technologies, evaluate information, organize information for practical use, integrate new information with existing knowledge, and use information for critical thinking and problem-solving (Neely, 2002, as cited in Akkoyunlu and Yılmaz, 2005).

In conclusion, there is a relationship between the concepts of “knowledge” and “innovation.” As noted, it is meaningful to acquire knowledge only if it is assimilated and transformed into innovation. Furthermore, it is argued that knowledge production is a prerequisite for innovation (Demirel and Seçkin, 2008). In other words, knowledge is necessary for innovation to take place, and innovation is necessary for creating new knowledge. When the concepts of innovation and knowledge are considered from the perspective of the education system, developments can be put into practice through teachers’ positive attitudes toward innovations, as well as their behaviors in accepting and utilizing innovations (Memmedova and Seferoğlu, 2014). Thus, as information is rapidly generating and expanding to enormous amounts in this digital age, it is essential for teachers to have the ability to acquire knowledge and access information. Indeed, both individual innovation and information literacy skills play a crucial role in training teachers who can keep pace with the changing conditions, needs, and requirements of the 21st century.

Based on the aforementioned information, pre-service teachers should generate new knowledge and utilize new technologies for achieving sustainable education goals (Cai et al., 2020; Lattu and Cai, 2020; Sonetti et al., 2019). These innovative teachers play a key role in promoting sustainable development in society (Acosta-Prado et al., 2020). Pre-service teachers not only help foster social innovation and development but also play a leading role in shaping tomorrow’s knowledge and technology initiatives (Reichert, 2019). The innovation performance of pre-service teachers plays a catalytic role in achieving the Sustainable Development Goals (SDG), particularly SDG 4, “Quality Education” (Žalėnienė and Pereira, 2021). For this reason, it is crucial to investigate the factors influencing pre-service teachers’ innovation performance and develop their innovative abilities in order to achieve sustainable education goals. However, only a limited number of studies in the relevant literature have focused on the development of pre-service teachers’ digital skills, particularly on how these skills influence their innovation performance.

Finally, future-oriented thinking skills and interpersonal skills are seen as a key to the training of pre-service teachers in sustainable education (Brundiers et al., 2021). In addition, some researchers argue that self-awareness skills (self-reflection, self-assessment, and self-regulation) are key competencies for sustainable development (Brundiers and Wiek, 2017; United Nations Educational, Scientific and Cultural Organization (UNESCO), 2017; Wamsler et al., 2018). As noted in these discussions, knowledge transfer, application, and innovation skills have become increasingly crucial for supporting pre-service teachers’ innovation performance and personal development in the digital context (Pilav-Velić et al., 2021). However, this issue is rarely discussed in academic literature. Furthermore, online platforms serve as tools for online learning, and these tools are one of the key factors distinguishing online learning from traditional learning (Castro-Schez et al., 2021). Based on Biggs’s Presage-Process-Product (3P) learning model, this study posits that information literacy and online knowledge sharing process would have a significant impact on pre-service teachers’ innovation performance. Biggs’s Presage-Process-Product (3P) learning model provides a theoretical framework for addressing the research question in this study. It offers an illuminating overview of the fundamental elements of the learning process and their interrelationships. Biggs proposed three stages of learning: Presage, Process, and Product (Biggs, 1987). The presage involves factors such as student characteristics and the learning environment. The process encompasses the learning methods used by students in the learning process. Finally, the product refers to the learning outcomes. Related studies in the literature have used the 3P model to examine the relationship between students’ individual learning backgrounds including their cognitive level and subject interest (Lee and Chan, 2018), environmental factors such as their perception of teaching methods (Deng et al., 2019), process factors such as learning strategies and learning methods (Biggs and Moore, 1993), and outcomes such as academic performance and learning satisfaction (Barattucci et al., 2017).

Biggs (1987) argued that personal and environmental factors (Presage) can influence the learning process (Process) and, consequently, learning outcomes (Product). Although the 3P model is widely used in traditional learning environments, it has been applied in only a limited number of studies examining the online learning process of pre-service teachers. This study applies the 3P model to the online environment with the aim of examining the effect of information literacy skills on innovation skills recognized as important skills in the 21st century. Specifically, the Presage stage encompasses pre-service teachers’ information literacy skills and their perception of environmental factors of online learning platform, while the Process stage refers to their online knowledge sharing process. Finally, the Product stage represents pre-service teachers’ innovation performance.

The research hypothesis regarding the information literacy (presage) and innovation performance (product) is presented below.

Information literacy emerges as a significant prerequisite influencing online learning. Information literacy is divided into four dimensions including information awareness, information acquisition, information discrimination, and information application recognized as key skills in the digital age. In today’s digital age, information is becoming increasingly complex. As a result, it has become essential for students to develop the ability of selecting, evaluating, and managing information in order to engage in learning activities (Gómez-García et al., 2020). Information literacy skills are closely related to pre-service teachers’ innovation skills (Chang and Hsu, 2015). Individuals with a high level of information literacy can actively and effectively implement innovative practices and achieve more innovative outcomes by using available resources efficiently.

In today’s information societies, the success of education systems is directly linked to the level of 21st century skills acquired by pre-service teachers, who will educate future generations (Voogt and Roblin, 2010). The modern educational paradigm requires teachers not only to be traditional transmitters of knowledge, but also to be innovative professionals who can adapt to ever-changing educational dynamics (Aithal and Aithal, 2023; Cohen and Levinthal, 1990). Pre-service teachers’ information awareness in the light of educational and technological developments (H1a), high-quality information acquisition from academic and pedagogical sources (H1b), discrimination of information scientifically and ethically from the vast amount of data in digital environments (H1c), and ultimately application of this filtered information by incorporating it into concrete teaching processes and lesson plans (H1d) constitute the cornerstones of innovative practices in education. Although there are studies examining the relationships between information literacy, digital competencies, and 21st century skills, there are few studies that empirically examine the direct effect of the subdimensions of information literacy on pre-service teachers’ innovation performance. Accordingly, this study aims to fill a significant gap in the relevant literature by explaining the mechanisms linking information literacy to the innovation performance of future teachers. So, the following hypotheses were examined in this study.

*Hypothesis 1 (H1a):* Information awareness literacy positively influences innovation performance.

*Hypothesis 1 (H1b):* Information acquisition literacy positively influences innovation performance.

*Hypothesis 1 (H1c):* Information discrimination literacy positively influences innovation performance.

*Hypothesis 1 (H1d):* Information application literacy positively influences innovation performance.

The research hypothesis regarding the information literacy (presage) and the online knowledge sharing process (process) is presented below.

The concept of the online knowledge sharing process in this study differs from the compulsory online courses offered by higher education institutions. This concept refers to the online knowledge sharing outside the university curriculum. In other words, pre-service teachers carry out information processing, integration, and innovation activities through online learning methods (such as browsing, liking, sharing information, commenting, and other online learning exercises). In this context, information literacy reflects students’ ability to comprehend, internalize, express, and apply information (Association of College and Research Libraries, 2015). These skills encourage active learning by enabling pre-service teachers to transform tacit knowledge into explicit knowledge and share it through online learning platforms (Nonaka et al., 2006). However, Hemmati (2017) found that the impact of information literacy on the online knowledge sharing process differs from person to person. Indeed, while teachers’ information literacy skills were found to positively predict online knowledge sharing process, pre-service teachers’ information literacy skills were not significantly associated with knowledge sharing or knowledge application process (Hemmati, 2017). Based on these contradictory findings, it is of great importance to investigate the impact of pre-service teachers’ information literacy skills on online knowledge sharing process comprehensively.

In the digitalizing education ecosystem, pre-service teachers’ innovation performance is not limited to their individual classroom practices but is also shaped by their knowledge sharing behavior within professional learning communities (Admiraal et al., 2017; Vangrieken et al., 2015). In this context, the online knowledge sharing process refers to the collective exchange of pedagogical experiences, teaching materials, and innovative ideas among pre-service teachers via digital platforms, forums, and social networks (Chen and Zou, 2026). Being active participants on these digital platforms depends largely on individuals’ level of information literacy (H2). The literature of Social Exchange Theory and Knowledge Management asserts that individuals must be firstly confident in the credibility of the information they share and feel intellectually competent in order to be willing to share knowledge in online environments (Wang and Noe, 2010). Pre-service teachers with strong information literacy skill have a high level of confidence in producing reliable and innovative content on digital networks. Because they can discriminate the accuracy of the educational data by filtering it through critical lens. Consequently, pre-service teachers’ high level of information literacy positively fosters high-quality and innovative knowledge sharing process that supports peer learning (Ergül and Taşar, 2023). This situation clearly highlights the importance of developing both information literacy skills and online collaboration practices in teacher education curriculum. In light of this importance, the following hypothesis was examined.

*Hypothesis 2:* Information literacy positively influences online knowledge sharing process.

The research hypothesis regarding the online knowledge sharing process (process) and innovation performance (product) is presented below.

This study examines both quantitative and qualitative aspects of online knowledge sharing process (Chang et al., 2014). The first approach emphasizes the number and activity of posts rather than the quality of the knowledge, while the second approach focuses on the value and novelty of the shared knowledge. Findings have shown that online learning process (such as the online knowledge sharing) offers opportunities to gain various forms of knowledge and experiences. The internalization of knowledge from different fields, in turn, will enhance innovation performance (Nonaka et al., 2006; Nonaka, 2007). Huang (2007) found that both the quantity and quality of the online knowledge transfer have a significant impact on innovation performance. Compared to quantity-oriented methods, quality-oriented knowledge transfer methods can lead to higher innovation performance.

There is a close relationship between knowledge sharing via digital networks, educational outcomes, innovation, and the structural dynamics of the sharing process. In this regard, the literature generally addresses online knowledge sharing processes as two fundamental dimensions: Quantity-oriented and quality-oriented (Haas and Hansen, 2007; Iskoujina and Roberts, 2015). Pre-service teachers engage in quantity-oriented knowledge sharing (Qty-KSP) by participating in continuous, and intensive interactions within peer groups or digital platforms. This fosters the creation of a broad pool of ideas. Thereby, this situation positively influences pre-service teachers’ innovation performance (H3a) (Kankanhalli et al., 2005; Wasko and Faraj, 2005). The quantity of shared knowledge acts as a catalyst in the processes of designing creative materials and developing original teaching methods by increasing the possibility of confronting different perspectives (Chiu et al., 2006). On the other hand, the quality-oriented knowledge sharing process (Qlty-KSP) is defined as the depth, accuracy, educational value, and applicability of the shared knowledge and it is also a critical factor that directly enhances pre-service teachers’ innovation performance (H3b) (Wang and Noe, 2010). Verified, high-quality posts that meet pedagogical standards reduce the risk of pre-service teachers making mistakes in their classrooms or during microteaching sessions. They also provide pre-service teachers practical, innovative solutions that can be directly applied in the classroom (Chiu et al., 2006). Consequently, both the frequency (quantity) of innovative ideas shared on online platforms and the educational and scientific value (quality) of these ideas emerge as two complementary mechanisms that maximize future teachers’ capacity for pedagogical innovation. Based on this, the following hypotheses were tested in this study. Based on this, the following hypotheses were tested in this study.

*Hypothesis 3 (H3a):* Quantity-oriented online knowledge sharing process (Qty-KSP) has a positive impact on innovation performance.

*Hypothesis 3 (H3b):* Quality-oriented online knowledge sharing process (Qlty-KSP) has a positive impact on innovation performance.

The research hypothesis regarding the mediating role of online knowledge sharing processes is presented below.

Individuals with a high level of information literacy have a strong sense of information awareness. They can also actively participate in the knowledge sharing process by utilizing information and communication technologies. This interactive process helps to generate new ideas and insights regarding the dissemination, synthesis, assimilation, and transformation of knowledge. Also, this process fosters creativity and innovation performance (Sun et al., 2022). Jinadu and Kaur (2014) found that individuals with high levels of information literacy have more access to information sources and they are able to discern the effectiveness of information. At the same time, these individuals can construct knowledge by sharing it with others. As a result, they are able to gain more experience in innovation practices. This process will further develop individuals’ relevant skills and support their innovation performance (Sun et al., 2022). Furthermore, a study conducted by Yin (2018) found that information literacy and new technologies and methods directly influence creativity.

Based on all of the relational dynamics described above, pre-service teachers’ information literacy skills directly influence innovation performance. On the other hand, this process may be indirectly triggered through certain behavioral mechanisms. In this context, the quantitative (Qty-KSP) and qualitative (Qlty-KSP) dimensions of online knowledge sharing process serves as a critical bridging mechanism in this relationship (H4a and H4b). Social Cognitive Theory and the Absorptive Capacity perspectives argue that individual competencies (information literacy) must undergo active interaction and information process in order to be transformed into concrete outcomes (innovation performance). A pre-service teacher with a high level of information literacy does not keep the pedagogical knowledge solely to themselves (Chiu et al., 2006). By sharing this intellectual knowledge frequently (in terms of quantity) on digital platforms, they create a collective pool of innovation (H4a). Similarly, pre-service teachers’ information literacy enhances the scientific and pedagogical depth (in terms of quality) of the educational content they share within their peer networks. This high-quality knowledge transfer also directly fuels the pre-service teachers’ innovation performance in course design and material development (H4b). Consequently, online knowledge sharing process transforms the information literacy into dynamic and functional actions that enhance pre-service teachers’ innovation performance. It is of great importance for the strategic design of teacher training policies in the digital age to understand how information literacy skills pave the way (through a mediating mechanism) for innovative educational outcomes (such as innovation performance) by increasing both the quantity and quality of sharing within online networks (Sun et al., 2022). In line with this importance, the following hypotheses were tested in this study.

*Hypothesis 4 (H4a):* Quantity-oriented online knowledge sharing process (Qty-KSP) mediates the relationship between information literacy and innovation performance.

*Hypothesis 4 (H4b):* Quality-oriented online knowledge sharing process (Qlty-KSP) mediates the relationship between information literacy and innovation performance.

The research hypothesis regarding the environmental factors in online learning (presage) and innovation performance (product) is presented below.

In addition to students’ individual characteristics, learning environments (such as peer interaction, the atmosphere of discussion among peers, the instructional context, and positive interaction with teachers) are also key components of the predictive variables in the 3P model. Students’ perception and evaluation of the learning environment is linked to their learning process, and this process leads to different learning outcomes (Biggs, 1987). Peers can help one another and learn from each other as they work together to achieve common goals on the online learning platform. Positive peer effect encourages students to actively engage in the online knowledge sharing process and it is an important factor in fostering innovation performance (Van Popta et al., 2017). In addition, the online knowledge sharing process relies on an online learning platform. Indeed, platform support is a key environmental factor that influences students’ participation in the online knowledge sharing process and innovation performance (Sun et al., 2022). Based on this, this study examines the environmental factors perceived by students as a significant “Presage Factor.” This factor has two dimensions as the peer support and online platform support.

Pre-service teachers’ innovation performance is strongly shaped not only by their individual competencies but also by the external ecosystem in which they interact. This highlights the key role of environmental factors in innovation processes as noted in the literature (H5) (Amabile, 1997; Yeşil et al., 2012). Environmental factors (such as the institutional climate, technological infrastructure, faculty members’ innovative approaches, supportive attitudes, and peer culture) create a motivating environment for pre-service teachers to develop creative ideas and put them into practice (Scott and Bruce, 1994; Thanasi-Boçe and Hoxha, 2024). In line with the “environment-behavior-individual” interaction perspective of Social Cognitive Theory, a supportive educational environment directly enhances pre-service teachers’ innovation performance (Bandura, 1986). On the other hand, these environmental supports not only directly influence pre-service teachers’ innovation performance but also serve as an indirect mechanism by transforming their interaction patterns on digital platforms (H6a and H6b) (Aleksieva, 2025). An academic environment with innovation and knowledge production facilitates the establishment of a broad collective pool of ideas by prompting pre-service teachers to engage more frequently, intensely, and continuously on digital platforms (Qty-KSP) (H6a) (Wasko and Faraj, 2005). Similarly, environmental factors characterized by high-quality educational resources and a climate of critical thinking enhance the pedagogical and scientific depth (Qlty-KSP) of the online knowledge. This high-quality knowledge indirectly nurtures the pre-service teachers’ innovation performance in designing original materials (H6b) (Wang and Noe, 2010). Consequently, supportive environmental factors bridge the gap to innovative educational outcomes (such as innovation performance) by optimizing both the quantity and quality of online knowledge sharing process. It is of central importance in structuring modern and sustainable teacher education strategies. Based on this, the following hypotheses were tested in this study.

*Hypothesis 5:* Environmental factors have a positive impact on pre-service teachers’ innovation performance.

*Hypothesis 6 (H6a):* Quantity-oriented online knowledge sharing process (Qty-KSP) mediates the relationship between environmental factors and innovation performance.

*Hypothesis 6 (H6b):* Quality-oriented online knowledge sharing process (Qlty-KSP) mediates the relationship between environmental factors and innovation performance.

**Figure 1:**
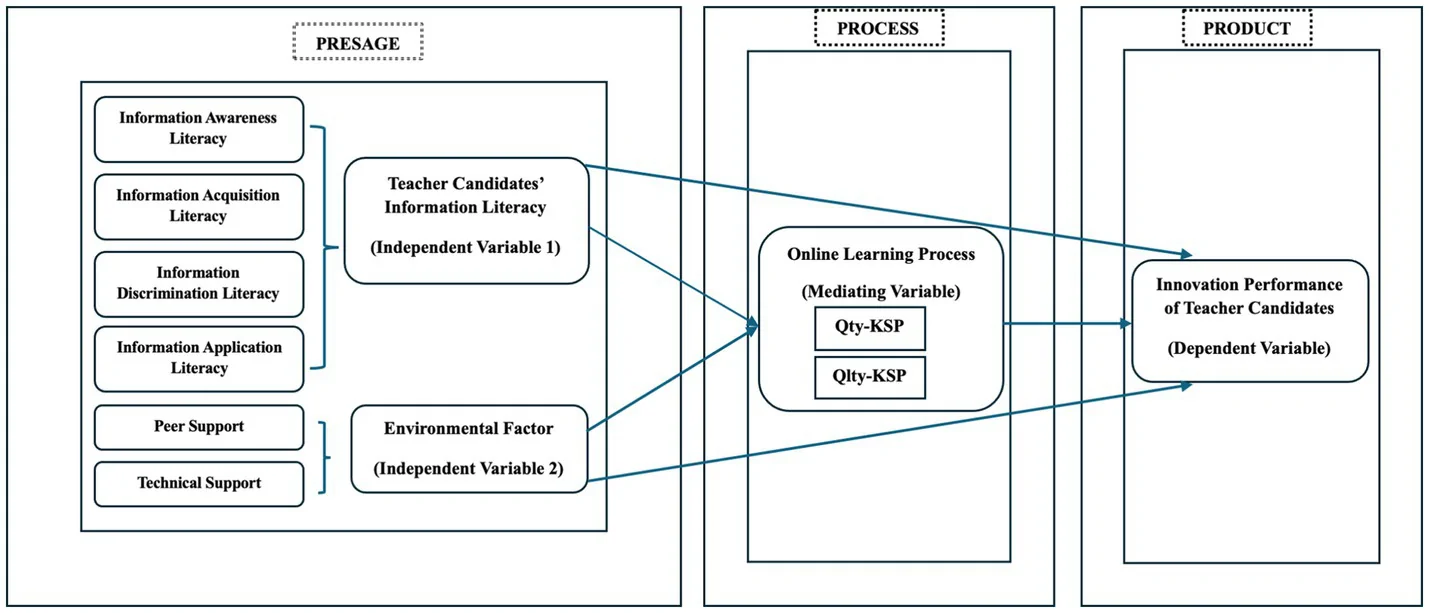
Process model of online-learning results based on 3P model. Adapted from Sun et al. (2022), Attribution 4.0 International (CC BY 4.0). Source: Retrieved from the https://www.mdpi.com/2071-1050/14/13/7789] Accessed Date: 21 March 2026.

In line with the conceptual framework and literature review presented above, this study proposes a theoretical model based on the 3P model (see Figure 1) regarding the factors influencing pre-service teachers’ innovation performance.

To explain the complex network of relationships illustrated in Figure 1, the theoretical framework of this study is based on Biggs’s (1987) Presage-Process-Product (3P) model. The 3P model argues that learning and performance outcomes do not develop in a linear pattern. Instead, it argues that a “product” emerges as a result of the integration of personal and environmental inputs (Presage) with the process of information management and interaction (Process) (Biggs and Moore, 1993). However, educational literature generally focuses on linear relationships between inputs and outputs, either neglecting the “process” stage or treating online knowledge sharing as a unidimensional or superficial phenomenon (Chiu et al., 2006). However, Biggs (1987) has noted that innovative and high-level outcomes are only possible through deep orientations. Accordingly, quantity of the knowledge sharing process (quantity-oriented process) and the depth, accuracy, and pedagogical value of the shared knowledge (quality-oriented process) enhances innovative outputs in different ways (Chiu et al., 2006; Wang and Wang, 2012). The relevant literature reveals that the effects of individual competencies and environmental factors on innovation performance are primarily examined within the context of corporate organizations, and industrial settings (Wang and Wang, 2012). However, little research has been conducted at the pre-service teachers, who will be the true pioneers of sustainable innovation and digital transformation in education systems. The pre-service teachers’ innovative tendencies have the potential to directly shape their teaching strategies in their professional lives and, consequently, the human resources of the future (Hsiao and Lin, 2023). Moreover, in the relevant literature, the concept of “environment” is generally examined through a unidimensional approach. The combined effect and interaction of systemic/technical capabilities (technological infrastructure) and social dynamics (peer support) are overlooked (Aparicio et al., 2016). While technological infrastructure cannot guarantee online interaction and innovation by itself, these technological tools become functional only within a social support ecosystem encouraging peers to share knowledge with one another (Lu et al., 2023).

Based on Biggs’s 3P model, this study examines the impact of personal inputs (information literacy) and socio-technical environmental inputs (online platform and peer support) on pre-service teachers’ innovation performance (Product) by modeling these factors together using quantitative and qualitative online knowledge sharing process (Process) as mediators. Traditional statistical methods lack the ability to holistically test this systemic, sequential, and multilayered within a single model (Kline, 2023). Therefore, this study utilized Structural Equation Modeling (SEM). The primary rationale for selecting the 3P (Presage-Process-Product) model in this study is the model’s ability to explain individual, environmental, and behavioral dynamics within the educational ecosystem as part of a holistic system. Although alternative models such as the Technology Acceptance Model (TAM) or the Unified Theory of Acceptance and Use of Technology (UTAUT) exist in the relevant literature, these models generally focus solely on users’ intention to adopt a technology and thus fail to adequately explain pedagogical and performance-based processes (Venkatesh et al., 2003). In contrast, Biggs’s (1987) 3P model aligns with the structure of this study by defining information literacy (an individual competency) as the “presage,” quantitative and qualitative online knowledge sharing behavior as the “process” and finally, innovation performance as the “product” stage. Furthermore, this model argues that inputs do not directly translate into outputs but gain meaning through the process stage (Biggs and Moore, 1993). This theoretical framework provides an ideal foundation for testing the mediating role in this study. In conclusion, this study aims to fill a significant theoretical, empirical, and methodological gap by adapting Biggs’s 3P model to the teacher education in the digital information age. In summary, this study aims to fill a significant gap in the relevant literature by holistically and structurally explaining the mechanisms linking future teachers’ individual abilities, environmental inputs, online knowledge sharing dynamics, and pedagogical innovation capacities through Biggs’s theoretical model.

## 2 Method

### 2.1 Research group

In selecting the pre-service teachers, the “convenience sampling” technique was employed, as it involved individuals who were easily accessible, lived in the surrounding area, and volunteered to participate in this study. This technique was chosen to minimize the loss of time, money, and manpower (Büyüköztürk et al., 2016). The inclusion criteria include *(i) being actively enrolled in a faculty of education, (ii) actively using at least one online platform (learning management systems, academic forums, education-focused social media groups, etc.) for the purpose of sharing knowledge (quantitative and qualitative), (iii) having experience accessing and using digital information resources (databases, e-libraries, academic search tools), (iv) having access to an institutional/environmental infrastructure (university resources, internet access, etc.) that supports distance education, hybrid education, digital research, knowledge acquisition, and online learning or collaboration process, (v) having active access to the online learning management systems (LMS), digital databases, or internet infrastructure provided by the university or faculty,* and finally *(vi) actively using social/digital networks to share knowledge, and collaborate with peers in their academic or professional development process*. The inclusion criteria were applied as a methodological obligation to maintain the study’s scope and control for other variables. However, these criteria resulted in a potential risk of selection or sampling bias by excluding a specific part of the target population from the sample. Also known as self-selection bias, this situation may have resulted in the sample being dominated by individuals who were interested in the subject, access to the necessary technological infrastructure, and willing to complete the survey. Individuals with low digital literacy or limited internet access may not have been adequately represented in the sample. This situation restricts the study’s external validity and the generalizability of the findings to the population. However, the strict application of these criteria ensured that participants had homogeneous and direct experience with the research topic, thereby enhancing the study’s internal validity and measurement quality.

Finally, this study was conducted with a total of 503 students, of whom 302 (60.04%) were female and 201 (39.96%) were male. These participants were generally in the 18–25 age group (97.2%). Also, the educational level of their mothers and fathers was elementary school (39.2 and 27.4%), the type of high school they graduated from was Anatolian high school (74.1%), their department was guidance and psychological counseling (82.6%), their grade levels were second and third grades (35.8%), their GPA was 3.0 or higher (69.3%), their internet access was limited (66%), the amount of time they spent using the internet and information technologies was between 1 and 5 h (76.4 and 76.9%), the source they used to access information was digital/electronic/virtual (32.5%), and the information technology tools they used were computers and smartphones (50.5%).

### 2.2 Research model

Based on Biggs’s Presage-Process-Product model, this study examines the direct and indirect paths between information literacy, the online knowledge sharing process, and innovation performance by using structural equation modeling (SEM) and correlational survey model. Structural equation modeling allows for the testing of complex, multi-variable models and examination of the direct and indirect effects of the variables in the model (Creswell, 2012; Fraenkel and Wallen, 2009).

### 2.3 Data collection tools

The data collection tools were described below.

#### 2.3.1 Personal information form

The personal information form includes a total of 13 questions regarding the gender, age, parents’ educational background, type of high school graduated from, educational department, grade level, grade points average (GPA), sources used to access information, internet access, average daily internet usage time, information technologies, and the average daily usage time of these technologies.

#### 2.3.2 Information Literacy Scale

As part of the study, the “Information Literacy Scale” developed by Adıgüzel (2011) was used to determine the information literacy skill levels of pre-service teachers. Participants indicate their views regarding the processes of acquiring and structuring information by selecting one of the options: “Always,” “Most of the Time,” “Sometimes,” “Occasionally” and “Never.” The structural validity of the 29-item Information Literacy Scale was tested using the Maximum Likelihood (ML) estimation method in Mplus 8 software. In the initial Confirmatory Factor Analysis (CFA) model established with the scale’s original structure, the model fit indices were found to be below acceptable range (ℵ^2^/df = 4.91, RMSEA = 0.107, CFI = 0.72, TLI = 0.79). To refine the model, Mplus outputs were analyzed in detail. Accordingly, ten items with factor loadings below the 0.50, and five items that compromised the model fit by exhibiting excessive cross-loadings and high error covariances in modification indices, were progressively removed from the model. The analysis repeated with the remaining 14 items. Model fit indices were found to be above acceptable range (ℵ^2^/df = 1.87, RMSEA = 0.035, CFI = 0.95, TLI = 0.94), and the standardized factor loadings of the items ranged from 0.587 to 0.816. The Cronbach’s alpha internal consistency coefficient was calculated as 0.928, and the item-total test correlation values were found to range between 0.557 and 0.735. The Cronbach’s alpha value calculated as 0.926 in this study. These results demonstrate that the scale has high validity and reliability and it is a valid and reliable tool that can be used to determine pre-service teachers’ information literacy skill levels.

In order to assess the convergent validity of the scale, it was found that the Composite Reliability (CR) values ranged from 0.835 to 0.884, and the Average Variance Explained (AVE) values ranged from 0.562 to 0.612. Since the AVE values for all subscales exceeded the critically accepted value of 0.50 in the literature, the CR values exceeded the value of 0.70, and the CR > AVE was met for each dimension, the scale was found to have convergent validity (Hair et al., 2010). The Fornell-Larcker criterion was used to determine discriminant validity. The analysis revealed that the square root of the AVE value for each latent variable was higher than the correlation coefficients between that variable and the other variables. Furthermore, since the maximum shared variance (MSV) and average squared shared variance (ASV) values were found to be smaller than the AVE values, it was concluded that the sub-dimensions measure independent, distinct structures and discriminant validity was established.

#### 2.3.3 The Environmental Factor Scale

The Environmental Factor Scale was developed by the researcher. This scale includes peer and platform support subscales. The peer support subscale includes questions, such as “the platform gathers a group of members with rich professional knowledge and skills” and “in the process of discussion, members use understandable communication modes” and the platform support subscale includes questions, such as “I think the system of the network sharing platform is reliable” and “I think the network sharing platform is useful.” In order to determine the construct validity of the scale, as suggested in the relevant literature, it is necessary to first conduct an Exploratory Factor Analysis (EFA) and then a Confirmatory Factor Analysis (CFA) on another sample with similar characteristics. However, taking into account the time factor and financial constraints (Demir et al., 2020; Koçtürk and Kızıldağ, 2018), the data were divided into two random subgroups. EFA was conducted on the first group (153 participants) and CFA on the other group (153 participants). The standardized factor loadings of the items ranged from 0.576 to 0.823. In addition, scale’s ℵ^2^/df (4.70) are less than 5, CFI (0.96), TLI (0.95), RMSEA (0.076) and SRMR (<0.08) are almost within the acceptable range, which proved that the subscales have good validity. The Cronbach’s alpha of the scale is 0.909 which indicates the scale has high reliability.

In order to assess the convergent validity of the scale, it was found that the Composite Reliability (CR) values ranged from 0.826 to 0.879, and the AVE values ranged from 0.545 to 0.608. Since the AVE values for all subscales exceeded the critically accepted value of 0.50 in the literature, the CR values exceeded the value of 0.70, and the CR > AVE was met for each dimension, the scale was found to have convergent validity (Hair et al., 2010). The Fornell-Larcker criterion was used to determine discriminant validity. The analysis revealed that the square root of the AVE value for each latent variable was higher than the correlation coefficients between that variable and the other variables. Furthermore, since the MSV and ASV values were found to be smaller than the AVE values, it was concluded that the sub-dimensions measure independent, distinct structures and discriminant validity was established.

#### 2.3.4 Individual Innovation Scale

In order to assess individuals’ levels of innovation, Kılıçer and Odabaşı (2010) adapted the “Individual Innovation Scale,” originally developed by Hurt et al. (1977) into Turkish. Participants indicate their views related to individual innovation for each item by selecting one of the following options: “Strongly Agree,” “Agree,” “Neutral,” “Disagree,” and “Strongly Disagree.” The structural validity of the 20-item Individual Innovativeness Scale was tested using the Maximum Likelihood (ML) estimation method in Mplus 8 software. In the initial Confirmatory Factor Analysis (CFA) model established with the scale’s original structure, the model fit indices were found to be below acceptable range (ℵ^2^/df = 4.62, RMSEA = 0.115, CFI = 0.82, TLI = 0.80). To refine the model, Mplus outputs were analyzed in detail. Accordingly, four items with factor loadings below the 0.50, and seven items that compromised the model fit by exhibiting excessive cross-loadings and high error covariances in modification indices, were progressively removed from the model. The analysis repeated with the remaining 9 items and these items demonstrated an acceptable model fit (ℵ^2^/df = 1.82, RMSEA = 0.045, CFI = 0.97, TLI = 0.96), and the standardized factor loadings of the items ranged from 0.585 to 0.814. The scale’s internal consistency coefficient was calculated as 0.82, and its test–retest reliability as 0.87. The Cronbach’s alpha value was 0.781 in this study. These results demonstrate that the scale is a valid and reliable tool for determining pre-service teachers’ levels of innovation.

In order to assess the convergent validity of the scale, it was found that the Composite Reliability (CR) values ranged from 0.811 to 0.858, and the Average Variance Explained (AVE) values ranged from 0.588 to 0.603. Since the AVE values for all subscales exceeded the critically accepted value of 0.50 in the literature, the CR values exceeded the value of 0.70, and the CR > AVE was met for each dimension, the scale was found to have convergent validity (Hair et al., 2010). The Fornell-Larcker criterion was used to determine discriminant validity. The analysis revealed that the square root of the AVE value for each latent variable was higher than the correlation coefficients between that variable and the other variables. Furthermore, since the MSV and ASV values were found to be smaller than the AVE values, it was concluded that the sub-dimensions measure independent, distinct structures and discriminant validity was established.

#### 2.3.5 The Online-Learning-Process Scale

The Online-Learning-Process Scale was developed by the researcher. This scale includes Qlty-KSP and Qty-KSP subscale. The sample items included such as “the knowledge I participated in online sharing is reliable” and “the knowledge I participated in online sharing is complete.” In order to determine the construct validity of the scale, as suggested in the relevant literature, it is necessary to first conduct an EFA and then a CFA on another sample with similar characteristics. However, taking into account the time factor and financial constraints (Demir et al., 2020; Koçtürk and Kızıldağ, 2018), the data obtained from the research group were divided into two random subgroups. EFA was conducted on the first group (151 participants) and CFA on the other group (151 participants). The standardized factor loadings of the items ranged from 0.566 to 0.819. In addition, scale’s ℵ2/df (2.80) are less than 5, CFI (0.97), TLI (0.96), RMSEA (0.060) and SRMR (<0.08) are almost within the acceptable range, which proved that the subscales have good validity. The Cronbach’s alpha of the scale is 0.906 which indicates the scale has high reliability.

In order to assess the convergent validity of the scale, it was found that the Composite Reliability (CR) values ranged from 0.814 to 0.892, and the Average Variance Explained (AVE) values ranged from 0.538 to 0.627. Since the AVE values for all subscales exceeded the critically accepted value of 0.50 in the literature, the CR values exceeded the value of 0.70, and the CR > AVE was met for each dimension, the scale was found to have convergent validity (Hair et al., 2010). The Fornell-Larcker criterion was used to determine discriminant validity. The analysis revealed that the square root of the AVE value for each latent variable was higher than the correlation coefficients between that variable and the other variables. Furthermore, since the MSV and ASV values were found to be smaller than the AVE values, it was concluded that the sub-dimensions measure independent, distinct structures and discriminant validity was established.

Table 1 provides information regarding the measurement instruments.

**Table 1:** Information regarding the measurement instruments.

Scale	KMO	Chi-Square Value of Bartlett Test	Cronbach’s alpha	ℵ2/df	CFI	TLI	RMSEA	Factor loadings
Information Literacy Scale	0.953	7137.975 (*p* < 0.001)	0.926	1.87	0.95	0.94	0.035	0.587 to 0.816
The Environmental Factor Scale	0.934	2313.606 (*p* < 0.001)	0.909	4.70	0.96	0.95	0.076	0.576 to 0.823
Individual Innovation Scale	0.935	3196.057 (*p* < 0.001)	0.781	1.82	0.97	0.96	0.045	0.585 to 0.814
The Online-Learning-Process Scale	0.871	2754.473 (*p* < 0.001)	0.906	2.80	0.97	0.96	0.060	0.566 to 0.819

### 2.4 Data analysis

The aim of this study is to examine the relationship between information literacy, environmental factors, the online knowledge sharing process, and innovation performance. So, this study was examined the mediating role of the online knowledge sharing process in the effect of students’ information literacy on innovation performance. In this study, information literacy was identified as the first independent variable, environmental factors as the second independent variable, and innovation performance as the dependent variable. A structural equation model (Model 1) was constructed using Mplus 8 to examine the effects of information literacy and environmental factors on innovation performance. Additionally, Model 2 was created by adding a mediating variable to Model 1. Consequently, data analysis was conducted in two stages to test the conceptual frameworks proposed in this study. In the first stage (Model 1), the direct effects of information literacy and environmental factors on pre-service teachers’ innovation performance were examined. After confirming these direct relationships, the model was transformed into Model 2 by including online knowledge sharing process as a mediating variable. Through this mediating framework, the total effects of the independent variables on innovation performance were distinguished as direct and indirect pathways (effects). Item deletion was performed to improve the model fit of both Model 1 and Model 2. In this context, while 14 of the 29 items from the Information Literacy Scale were used, 9 of the 20 items from the Individual Innovation Scale were used in the model. According to Anderson and Gerbing (1988) relating or deleting indicators from the model are the preferred basic ways to ensure fitness of the model. Thus, item deletion and adding a new path indicator are the appropriate ways to get a satisfactory fit model.

## 3 Results

### 3.1 The impact of information literacy and environmental factor on innovation performance

**Figure 2:**
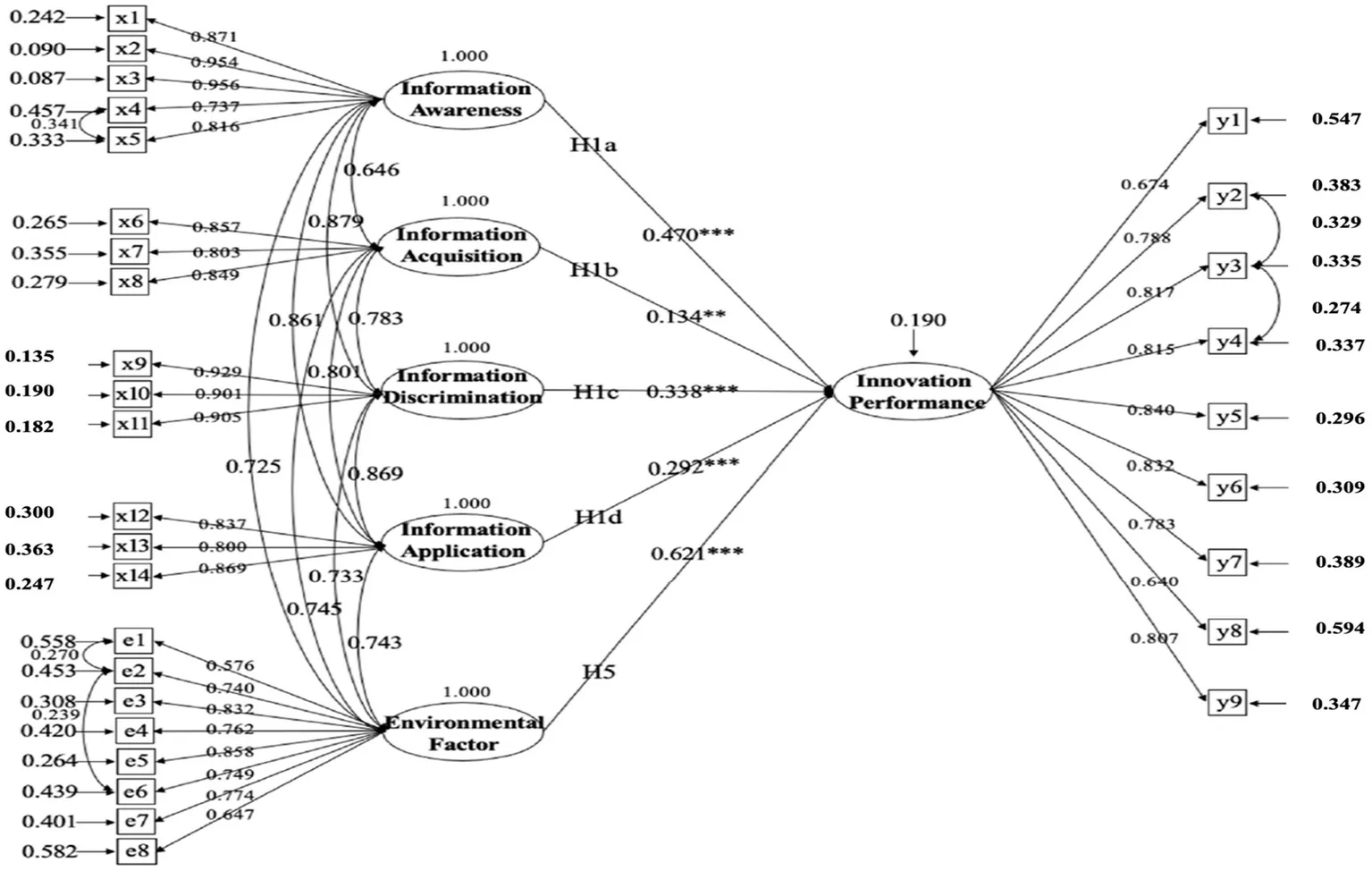
The impact of information literacy on innovation performance. **p < 0.05; ***p < 0.01. Adapted from Sun et al. (2022), Attribution 4.0 International (CC BY 4.0). Source: Retrieved from the https://www.mdpi.com/2071-1050/14/13/7789] Accessed Date: 21 March 2026.

In this study, which utilizes structural equation modeling, information literacy was identified as the first independent variable, environmental factors as the second independent variable, and innovation performance as the dependent variable. The model fit indices for Model 1 (ℵ^2^ (414) = 1211.8581**, ℵ^2^/df = 2.9272 < 5, RMSEA = 0.0621, CFI = 0.9311, TLI = 0.9231, SRMR = 0.0621) were found to be within an acceptable range. When Figure 2 is examined, information acquisition, awareness, discrimination, application literacy, and environmental factors related to online learning platforms were found to positively predict innovation performance at the 0.01 significance level. Compared to information application literacy (*γ* = 0.292, *p* < 0.01), information awareness (γ = 0.470, *p* < 0.01) and discrimination literacy (γ = 0.338, *p* < 0.01) have a more significant effect on innovation performance. In addition, environmental factors (such as peer and platform support) have a significant positive impact on innovation performance (γ = 0.621, *p* < 0.01). Peers with a shared vision actively participate in the online knowledge sharing process, while an efficient and stable platform accelerates the dissemination of effective knowledge. A high-quality online learning environment helps pre-service teachers to acquire knowledge effectively, foster innovative behavior, and increase innovation output. Based on the research findings above, hypotheses H1a, H1c, H1d, and H5 were supported, while hypothesis H1b was not confirmed.

### 3.2 Mediating effect test of online knowledge sharing behavior

**Figure 3:**
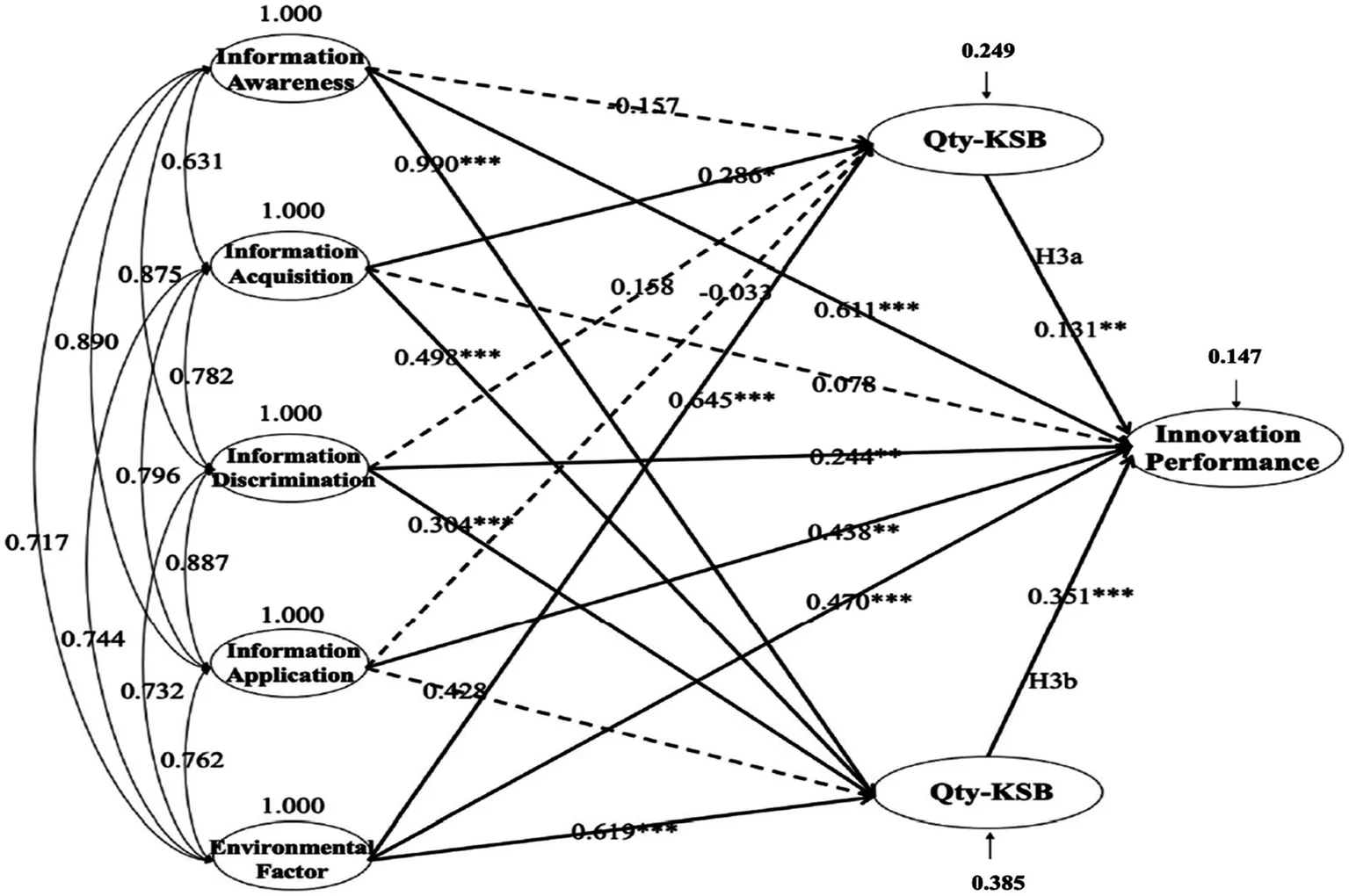
The impact of information literacy, environmental factors, and learning process on innovation performance. **p < 0.05; ***p < 0.01. Adapted from Sun et al. (2022), Attribution 4.0 International (CC BY 4.0). Source: Retrieved from the https://www.mdpi.com/2071-1050/14/13/7789] Accessed Date: 21 March 2026.

After adding the mediating variables “Qty-KSP” and “Qlty-KSP” to Model 1, Model 2 was created to reveal the effects of information literacy and environmental factors on innovation performance (see Figure 3). It had been determined that the model fit values were within an acceptable range. (ℵ^2^(707) = 2219.8321**, ℵ^2^ /df = 3.1401 < 5, RMSEA = 0.0652, CFI = 0.9171, TLI = 0.9081, SRMR = 0.0672). As shown in Figure 3, pre-service teachers with high levels of information awareness (γ = 0.990, *p* < 0.01), acquisition (γ = 0.498, *p* < 0.01), and discrimination literacy (γ = 0.304, *p* < 0.05) prefer the Qlty-KSP, whereas information application literacy (γ = 0.428, *p* > 0.01) does not have a statistically significant effect on the Qlty-KSP. With the exception of information acquisition literacy (γ = 0.286, *p* < 0.1), there is no direct relationship between the other information literacy skills and Qty-KSP. H2 has deviation. Furthermore, compared to Qty-KSP (γ = 0.131, *p* < 0.05), Qlty-KSP has a more obvious positive effect on innovation performance (γ = 0.351, *p* < 0.01). Hypotheses H3a and H3b are confirmed.

This study tests the mediation effect of online knowledge sharing process based on bootstrap. After repeatedly sampling 5,000 bootstrap samples for estimation, Qlty-KSP (M1) plays a partial mediating role in the relationship between information awareness (X1), acquisition (X2), discrimination (X3), application literacy (X4) and innovation performance (Y). In other words, when Qlty-KSP was added into the model as a mediating variable, the direct effects of independent variables on innovation performance remained statistically significant. Each dimension of information literacy has a positive impact on innovation performance, both directly and indirectly through a high-quality knowledge sharing process. In this model, there are four indirect paths as A1, B1, C1, and D1. For example, path A1 represents X1 → M1 → Y. Hypothesis H4b has been confirmed. A part of the effect of information literacy on innovation performance is mediated by Qlty-KSP, which accounts for 42.752% of the total effect.

Additionally, Qty-KSP (M2) plays a partial mediating role in the relationship between information literacy and innovation performance. When Qlty-KSP was added into the model as a mediating variable, the direct effect of information literacy on innovation performance remained statistically significant. There are four indirect paths as A2, B2, C2, and D2. Path D2 represents X4 → M2 → Y, with an effect size of 1.922%, and the mediating coefficient test is significant. However, since the 95% confidence intervals for the other three indirect paths include zero, the mediating effect is not significant, and H4a is partially supported. Other indirect paths were given in Table 2. Furthermore, the results from Model 2 indicate that environmental factors (such as stable, safe, and efficient online learning platforms) as well as peers with a common language, shared vision, and mutual trust significantly predict online knowledge sharing process. Qlty-KSP plays a partial mediating role between environmental factors and innovation performance (Path E1). The direct effect of environmental factors on innovation performance remained statistically significant even after the Qlty-KSP was included in the model. The mediation coefficient is 36.431%, and the 95% confidence interval [0.2832, 0.4371] does not include zero. Therefore, H6b has been supported. On the other hand, there is no significant mediating effect of Qty-KSP between environmental factors and innovation performance (Path E2). The mediating effect coefficient is −0.006. Since the 95% confidence interval [−0.0302, 0.0201] includes zero, Hypothesis H6a has not been confirmed. Finally, when compared to the Qty-KSP variable, Qlty-KSP is a stronger mediating variable and plays a positive mediating role in the effect of information literacy and environmental factors on innovation performance.

**Table 2:** Total effect and details of each mediation path.

Pathways	Mediation analysis	Effect	BootSE	95% confidence interval		*t*-value	*p*-value
				BootLLCI	BootULCI		
X1 → M → Y	Total effect	0.5101	0.0271	0.4571	0.5633	18.7731	0.000
	Direct effect	0.2943	0.0271	0.2422	0.3461	11.0862	0.000
	Indirect effect A1	0.2182	0.0231	0.1753	0.2641	X1 → M1 → Y	
	Indirect effect A2	−0.0021	0.0063	−0.0141	0.0102	X1 → M2 → Y	
X2 → M → Y	Total effect	0.8324	0.0362	0.7611	0.9034	23.0291	0.000
	Direct effect	0.4542	0.0401	0.3762	0.5324	11.4562	0.000
	Indirect effect B1	0.3822	0.0363	0.3143	0.4541	X2 → M1 → Y	
	Indirect effect B2	−0.0041	0.0122	−0.0282	0.0192	X2 → M2 → Y	
X3 → M → Y	Total effect	0.5342	0.0241	0.4871	0.5813	22.2713	0.000
	Direct effect	0.3222	0.0254	0.2731	0.3713	12.9833	0.000
	Indirect effect C1	0.2051	0.0222	0.1642	0.2492	X3 → M1 → Y	
	Indirect effect C2	0.0071	0.0053	−0.0032	0.0202	X3 → M2 → Y	
X4 → M → Y	Total effect	0.7803	0.0363	0.7093	0.8511	21.5001	0.000
	Direct effect	0.4372	0.0351	0.3684	0.5072	12.4072	0.000
	Indirect effect D1	0.3282	0.0342	0.2643	0.3974	X4 → M1 → Y	
	Indirect effect D2	0.0151	0.0071	0.0031	0.0303	X4 → M2 → Y	
X5 → M → Y	Total effect	0.9801	0.0342	0.9142	1.0473	28.8571	0.000
	Direct effect	0.6293	0.0443	0.5421	0.7162	14.2541	0.000
	Indirect effect E1	0.3574	0.0404	0.2832	0.4371	X5 → M1 → Y	
	Indirect effect E2	−0.0064	0.0132	−0.0302	0.0201	X5 → M2 → Y	

## 4 Discussion

### 4.1 Strengthening information literacy helps to increase pre-service teachers’ innovation performance

Information literacy has become the essential skill for training individuals about sustainable development in the digital age. Developing the information literacy of pre-service teachers is of great importance for enhancing their innovation performance and fostering sustainable higher education environments (Sun et al., 2022). This study was found that pre-service teachers’ information awareness, discrimination, and application literacy as well as information literacy significantly and positively predict innovation performance. In the digital age, the content of information is complex and there are many ways to access this information. Today, accessing information does not appear to be a key factor in increasing pre-service teachers’ participation in innovation activities. However, it is important for pre-service teachers to comply with ethical standards in order to carry out innovative activities in the information society. In addition, pre-service teachers can effectively assess the content and quality of information from various sources and construct their own knowledge based on complex information. These abilities help to foster critical thinking and practical skills. It also serves as a source of inspiration for more innovative behaviors and outcomes (Chang and Hsu, 2015). This finding is consistent with the results of previous studies, such as Gómez-García et al. (2020) and Chang and Hsu (2015). Furthermore, these studies have found that pre-service teachers’ ability to effectively acquire, evaluate, and manage information has become a key prerequisite for their participation in learning activities in the digital age, and this is closely linked to innovation performance.

In this study, “enhancing information literacy helps increase pre-service teachers’ innovation performance” is consistent with empirical findings in the contemporary teacher education literature (Odularu and Bokwe, 2025). This relationship can be theoretically grounded through Biggs’s (1987) 3P (Presage-Process-Product) model, which explains learning process as a holistic system. According to Biggs’s approach, overall success correlates with the quality of the inputs (Biggs and Tang, 2011). Within this theoretical framework, information literacy corresponds to the “presage” stage, the first stage of Biggs’s model. Information literacy is the most fundamental personal skill that determines pre-service teacher’s ability to access information, critically evaluate data, verify its accuracy, and reorganize information to meet a specific purpose. The literature has empirically demonstrated that teachers’ levels of information literacy directly fuels their cognitive flexibility, awareness of digital transformation, and research performance (Mohammed et al., 2024; Orhan Özen and Mazman Akar, 2026). Experimental studies have shown that structured information literacy interventions significantly improve pre-service teachers’ ability to critically evaluate information and problem-solving process (Chaliha et al., 2024; Yurdakal, 2024). Similarly, empirical studies conducted on faculty members and pre-service teachers confirm that information literacy positively predicts teachers’ innovative teaching behaviors (Trixa and Kaspar, 2024). Empirical evidence shows that educators with high information literacy experience creative self-efficacy. As a result, they demonstrate significantly higher levels of pedagogical innovation in their classrooms (Gong, 2023).

In Biggs’s (1987) 3P model, this strong intellectual foundation at the presage stage triggers the pre-service teacher to adopt a deep learning approach during the process of structuring knowledge. A pre-service teacher with high information literacy does not merely ignore the pedagogical challenges or technological innovations. Rather, they explore the source of the information and analyze it in depth. In this way, pre-service teachers gain a high level of self-confidence in moving beyond traditional templates, taking risks, and trying alternative methods in educational processes. As a natural consequence of this in-depth information processing, the level of innovation is maximized during the “product” stage, the final stage of Biggs’s model. According to Biggs’s principle of constructive alignment, the quality of innovation performance in the product stage is closely tied to the quality of information literacy in the presage stage (Biggs and Tang, 2011). Pre-service teachers with strong information literacy move beyond their role as passive practitioners who merely consume existing static knowledge. They become leaders who develop original digital materials in their classrooms, integrate modern teaching strategies into their lessons, and trigger innovative process in education (Naveed et al., 2023). In conclusion, from the perspective of Biggs’s 3P model, information literacy serves as a strategic catalyst by improving the quality of inputs (teacher education system), deepening the pre-service teacher’s cognitive process, and ultimately fostering the desired innovation performance in education.

### 4.2 Effective and efficient online learning environments help to increase pre-service teachers’ innovation performance

Digital transformation is becoming an indispensable part of people’s lives (Căpușneanu et al., 2021). Online learning environments are shifting from face-to-face interaction to hybrid interaction (Angouri, 2021). For this reason, the stability and effectiveness of the online learning platform, along with the positive peer influence of online platform users, have specific effects on pre-service teachers’ innovation performance (Tang et al., 2024). Furthermore, this study found that the Qtly-KSP variable acts as a mediator in the relationship between environmental factors and pre-service teachers’ innovation performance. Based on this finding, it can be concluded that an effective online platform can promote a higher-quality online learning experience for pre-service teachers and support their innovation performance. Finally, the two dimensions of peer support and platform support complement each other. A positive communication network and atmosphere of sharing, combined with the platform’s stable system structure, encourages students to participate in the interchange of ideas (Akram et al., 2021). At the same time, platform users’ active sharing behavior, combined with high-quality and effective content and an open discussion atmosphere, can enhance the platform’s effectiveness and create a positive learning environment. This finding demonstrates that the two dimensions are inseparable. The online learning platform and environmental factors work together to increase pre-service teachers’ innovation performance.

In this study, “effective and efficient online learning environments help increase pre-service teachers’ innovation performance” is consistent with numerous empirical findings in the literature related to digital pedagogy and modern teacher education (Ramos et al., 2025). Effective and efficient online learning environments correspond to the contextual elements (situational/teaching context) in the “presage” stage. As a direct result of this well-structured process, pre-service teachers’ innovation performance is maximized in the “product” stage. According to Biggs’s principle of constructive alignment, the quality of innovation performance in the product stage is closely tied to the quality of the learning environment in the presage stage (Biggs and Tang, 2011). A user-friendly and efficient online interface reduces the cognitive overload of pre-service teachers, making it easier for them to focus on self-regulated online learning, collaborative inquiry, and metacognitive strategies (Butar et al., 2024). Indeed, Khlaisang and Teo (2024) and Jiang et al. (2023) have empirically reported that pre-service teachers demonstrate significantly higher engagement in online knowledge sharing process and peer interaction at the effective digital environments compared to traditional environments. In this productive process, supported by social interaction and rich digital materials, pre-service teachers restructure their own mental schemas by personally experiencing how to integrate technology into educational process.

Empirical studies have shown that well-structured digital learning environments with a high level of interaction are the most critical external input for fostering pre-service teachers’ technological pedagogical content knowledge (TPACK), digital readiness, and perceptions of self-efficacy (Butar et al., 2024; Tondeur et al., 2020). For example, a longitudinal study conducted by Singh et al. (2021) found that well-designed online learning platforms increase students’ academic motivation and their willingness to adopt technology. Similarly, an empirical study conducted by Al-Ansi et al. (2023) in higher education confirms a strong and positive relationship between the effectiveness of digital infrastructure and students’ creative problem-solving skills. It has been found that enriched online learning experiences positively predict individuals’ innovative teaching behaviors, creative self-efficacy, and proactive work approaches (Qi et al., 2026). Furthermore, in a study by Jo (2024), it was empirically demonstrated that the quality of the online environment has a direct impact on teachers’ performance in integrating innovative technologies. Pre-service teachers demonstrate significantly higher levels of innovation, moving beyond traditional templates, taking risks, and developing original digital pedagogical strategies in the effective digital ecosystem (Kohnke and Zou, 2026).

### 4.3 High-quality online knowledge sharing process helps to increase pre-service teachers’ innovation performance

Various online learning process has different effects on pre-service teachers’ innovation performance (Razmerita et al., 2020). These findings are consistent with the results of the study conducted by Huang (2007). It is considered that pre-service teachers’ engagement in high-quality and sustainable online learning and collaboration process is a keyway to enhance their innovation performance. According to Biggs (1987), the process component of the 3P model suggests that different motivations lead to different learning behaviors and learning methods, which in turn influence learning outcomes (Deng et al., 2019). Qty-KSP indicates that pre-service teachers are motivated by external factors and hope to earn virtual currency or forum points through knowledge sharing (Jin et al., 2015). Online knowledge sharing process primarily involves the posting of numerous meaningless messages on online platforms and the accumulation of platform points or experience points by providing meaningless responses to discussion topics. Thus, the methods of conveying knowledge through Qty-KSP make the online learning process more practical. These conditions may have a negative impact on critical thinking, integration, and the reconstruction of knowledge, thereby contributing less to innovation performance. In contrast, Qlty-KSP represents a more in-depth approach to traditional learning. In this process, driven by social motivation and intrinsic self-motivation, more importance is given to the meaningful sharing and interchange of knowledge. These students explore the potential relevance and purpose of online learning tasks and compare knowledge from multiple perspectives. This in-depth online learning process is of great importance in terms of developing individuals’ capacity for sustainable development and fostering their innovation performance (Wasko and Faraj, 2005).

In this study, “knowledge sharing in high-quality online learning process increases pre-service teachers’ innovation performance” is consistent with numerous empirical findings in the teacher education literature (Camayang et al., 2023). Empirical studies have shown that flexible, easily accessible digital ecosystems with central resources, collaborative environments, and a strong pedagogical infrastructure are the most critical external factors supporting pre-service teachers’ innovation performance (Baysen et al., 2017). Pre-service teachers’ cognitive overload is reduced on user-friendly, enriched digital platforms without technical issues. Furthermore, these platforms enable them to focus on behavioral, cognitive, and social engagement process (Krooi et al., 2024).

According to Biggs’s principle of constructive alignment, the quality of innovation performance at the product stage is closely linked to the quality of the presage and process stage (Biggs and Tang, 2011). Al-Ansi et al. (2023) confirm that the infrastructure and pedagogical quality in digital environments are the strongest predictors of students’ creative performance. A large-scale empirical study conducted by Huang et al. (2024) found that high-quality online courses designed with innovative teaching strategies influence students’ innovation performance. Empirical studies conducted with pre-service teachers and teachers also clearly support this linear and indirect mechanism. It has been found that pre-service teachers’ innovative teaching behaviors and creative self-efficacy has significantly increased in the enriched online learning environment (Camayang et al., 2023). A study by Seifert and Bar-Tal (2023) found that a high-quality online learning environment and a sense of belonging to that environment enhance teachers’ innovative technology integration performance in the classroom. Pre-service teachers tend to be more flexible, bold, and open to taking risks when adapting complex technological tools (e.g., artificial intelligence and Web tools) to their lessons in a high-quality, interactive, and proactive digital ecosystem.

### 4.4 The online knowledge sharing process plays a mediating role in the impact of information literacy on the innovation performance

Higher level of information literacy and participation in high-quality online learning process has become essential prerequisites for pre-service teachers’ professional development in the digital age (Sun et al., 2022). According to Yin (2018), knowledge sharing plays a mediating role between information literacy and organizational innovation performance. This study found that the Qlty-KSP variable plays a partial mediating role in the positive effects of information literacy and environmental factors on innovation performance. This study found that the Qty-KSP variable acts as a mediator in the relationship between information application literacy and individual innovation. Pre-service teachers with high level of information literacy pay greater attention to the importance of knowledge sharing. Pre-service teachers with a high level of information literacy generally prefer to use Qlty-KSP rather than simply reposting content published by others. They summarize the main ideas and structure of the information, integrate theory and practice to form their own knowledge and perspectives, and share these. Stable, effective, and efficient platforms provide a robust foundation for advanced peer learning aligned with Qlty-KSP. Information literacy and environmental support has a significant positive impact on pre-service teachers’ innovation performance by fostering critical thinking and restructuring their knowledge systems. However, Qty-KSP stems from external motivation more than from a high level of information literacy. This mediating effect appears to be more pronounced among active users who have a strong ability to integrate theory and practice.

In this study, “the online knowledge sharing process plays a significant mediating role in the effect of information literacy on pre-service teachers’ innovation performance” is in line with models in the educational sciences literature (Naveed et al., 2023). This indirect and cyclical mechanism can be grounded in a theoretical framework through Biggs’s (1987) 3P (Presage-Process-Product) model, which explains the interactions among the components of educational ecosystems. In this theoretical framework, the pre-service teachers’ information literacy corresponds to the learner’s personal characteristics at the “presage” stage. Biggs’s (1987) model argues that personal characteristics at the presage stage must undergo an active process stage in order to be transformed into a concrete outcome.

The empirical literature shows that pre-service teachers with strong information literacy (presage) manipulate information flows, discussion forums, and digital material production processes in online environments in a much more proactive manner, similar to what Biggs refers to as the “deep learning approach” (Trixa and Kaspar, 2024). Individuals with higher information literacy filter knowledge in the digital environments. They build a high-level process of learning and knowledge sharing in online environments, both in terms of quantity and quality (Krooi et al., 2024). Therefore, information literacy is a potential strength. On the other hand, the online knowledge sharing process is an operational arena that transforms this strength into kinetic energy by allowing pre-service teachers to restructure their mental schemas through peer interaction (Iskoujina and Roberts, 2015). In the “product” stage, the pre-service teacher’s innovation performance reaches maximum levels. According to Biggs’s principle of constructive alignment, the quality of innovation performance in the product stage depends on the effective matching of presage quality with process quality (Biggs and Tang, 2011). Indeed, studies conducted on educators also empirically confirm this mediating mechanism. According to these studies, when individual competencies are combined with high-quality online learning experiences, digital engagement, and collaborative process, these factors translate into innovative teaching behaviors and creative self-efficacy (Lyu et al., 2026). In conclusion, when Biggs’s 3P model and the empirical literature are evaluated together, it cannot be expected that information literacy will directly lead to innovation performance. Information literacy (presage) enriches pre-service teachers’ online knowledge sharing process (process), and this process ultimately triggers the desired innovation performance in education system (product). This finding clearly demonstrates that the online knowledge sharing process plays a strategic bridging role in the transformation of information into innovation.

## 5 Conclusion

The meaning of sustainability in higher education has changed in the digital age. This study, based on Biggs’s Presage-Process-Product model, examines the direct and indirect paths of the relationship between information literacy, environmental factor, the online knowledge sharing process, and innovation performance. The findings highlight the importance of pre-service teachers’ innovation performance in promoting the sustainable development of higher education. This can be achieved by enhancing pre-service teachers’ information literacy and ensuring the quality of the online learning process. Furthermore, this approach could serve as a key pathway to achieving the “Quality Education” sustainable development goal and advancing the 2030 Agenda for Sustainable Development.

This study extends Biggs’s 3P learning model to the context of online learning in teacher education. It also focuses on practical issues such as the sustainable development of higher education and skill development in the digital age. In the digital age, information literacy and extracurricular online learning process is recommended as key pathways for improving the innovation skills of pre-service teachers and achieving sustainable development (Sun et al., 2022). Based on the findings of this study, it is necessary to provide an online learning process that fosters information literacy skills and an inclusive learning environment in order to support pre-service teachers’ sustainable development capabilities and achieve high-quality learning outcomes in the digital age. This finding supports everyone’s right to equitable access to digital services. It is believed that these results will contribute to the achievement of the Sustainable Development Goals, particularly “Goal 4: Quality Education.”

Based on the findings of this study, the following suggestions were recommended: (1) incorporating courses related to information literacy into the teacher education curriculum and focusing on developing pre-service teachers’ skills of information discrimination and application literacy, (2) encouraging pre-service teachers to actively participate in knowledge sharing process through out-of-class learning platforms such as virtual academic communities, and developing digital competencies for sustainable development through high-quality knowledge sharing practices and finally, and (3) building a user-friendly knowledge sharing platform for pre-service teachers and providing an effective environment to improve the sustainability, flexibility, and digitalization of higher education (Miceli et al., 2021). Based on the holistic mechanism and the findings of Biggs’s 3P model presented in this study, strategic stakeholders are expected to take on specific responsibilities to improve both the quality of inputs (presage) and the quality of the process (process) in the teacher education system. In this context, it is essential for curriculum developers to integrate proactive learning modules into the system by moving away from the static transfer of knowledge. Also, they should focus on fostering information literacy and critical data analysis skills in the teacher education curriculum. Information literacy’s higher-level components (such as big data analytics, the verification of open-access educational resources, intellectual property rights, and digital rights management) should be taught to pre-service teachers both theoretically and practically. To improve the quality of online knowledge sharing process, “Online Micro-Learning Communities” should be made an integral part of the curriculum. In these communities, pre-service teachers work collaboratively on learning management systems (LMS) and produce original digital course materials, case studies, and e-portfolios instead of completing traditional end-of-term assignments. Instead of merely expressing opinions on digital platforms (quantity), pre-service teachers should be evaluated based on their ability to ground the information in academic sources, verify the reliability of data, and critique disinformation (quality). As the architects of the system at the macro level, policymakers must update the teacher competency standards to meet the requirements of the digital age. They should make information literacy and innovation performance fundamental accreditation criteria at the national level. At the institutional level, university administrators need to continuously invest in high-quality online learning infrastructures (absence of technical difficulties, user-friendly, and highly interactive) through distance education centers. It is critically important to establish digital badge reward systems to encourage pre-service teachers to share knowledge both quantity and quality. Finally, lecturers, who are the direct facilitators of these processes in the classroom, must not allow pre-service teachers to become passive consumers of content on digital platforms. They must incorporate peer assessment systems into their courses to trigger pre-service teachers’ cognitive, behavioral, and social engagement in online environments. Lecturers should actively guide pre-service teachers to question the accuracy, pedagogical validity, and quality of information shared in online discussion forums. In summary, it is possible to train teachers with high innovation performance by maximizing the quality of input and process through the coordinated and constructive alignment of all stakeholders.

Although this study makes significant contributions to the literature by explaining the mechanisms linking pre-service teachers’ information literacy and innovation performance, it has certain structural and methodological limitations. The first methodological limitation of the study is that the data were collected using self-report scales based on the participants’ own perceptions and evaluations. This carries the risk that participants may have been influenced by social desirability bias. In future studies, mixed research methods can be used to increase data diversity and reliability. Second, this study is based on a correlational and cross-sectional research design. So, it is difficult to fully establish the causal relationships within the Presage-Process-Product cycle described in Biggs’s 3P model. Also, it prevents the tracking of how variables change over time. To gain a clearer understanding of the changes in the variables over time and their causal cycle, it is recommended that future studies should be conducted using longitudinal research designs. Furthermore, various psychological and demographic variables (such as pre-service teachers’ individual motivational orientations, technology anxieties, or epistemological beliefs) should be integrated into the model in order to enrich Biggs’ Presage stage. Another significant limitation of the study involves the sample structure and external validity. The study’s sample is limited to pre-service teachers from a specific geographic region or university. Consequently, the empirical findings may not fully reflect the dynamics of teacher populations in different geographic regions and institutional cultures. To ensure the external validity and generalizability of the findings, future studies should focus on different cultural ecosystems and cross-cultural samples. In addition, background variables (such as the pre-service teachers’ branches or socioeconomic status) were not controlled in this study. Future studies could investigate the applicability of this study to students from different genders, educational backgrounds, and family backgrounds.

## Data Availability

The raw data supporting the conclusions of this article will be made available by the authors, without undue reservation.
